# Clinical Validation of an Office-Based ^14^C-UBT (Heliprobe) for *H. pylori* Diagnosis in Iranian Dyspeptic Patients

**DOI:** 10.1155/2011/930941

**Published:** 2011-06-22

**Authors:** Fariborz Mansour-Ghanaei, Omid Sanaei, Farahnaz Joukar

**Affiliations:** ^1^Gastrointestinal and Liver Diseases Research Center (GLDRC), Guilan University of Medical Sciences, 41448-95655 Rasht, Iran; ^2^Department of Internal Medicine, Guilan University of Medical Sciences, 41448-95655 Rasht, Iran

## Abstract

*Background*. We encountered repeatedly, in our clinical practice, discordant results between UBT and histopathology about *H. pylori* infection. *Goal*. To study the diagnostic accuracy of Heliprobe ^14^C-urea breath test (^14^C-UBT) for detection of *H. pylori* infection in an Iranian population. *Study*. We enrolled 125 dyspeptic patients in our study. All of them underwent gastroscopy, and four gastric biopsies (three from the antrum and one from the corpus) were obtained. One of the antral biopsies was utilized for a rapid urease test (RUT), and three others were evaluated under microscopic examination. Sera from all patients were investigated for the presence of *H. pylori* IgG antibodies. The ^14^C-UBT was performed on all subjects using Heliprobe kit, and results were analyzed against the following gold standard (GS): *H. pylori* infection considered positive when any two of three diagnostic methods (histopathology, RUT, serology) are positive. *Results*. According to data analysis, the Heliprobe ^14^C-UBT had 94% sensitivity, 100% specificity, 93% negative predictive value (NPV), 100% positive predictive value (PPV), and 97% accuracy, compared with GS. *Conclusion*. The Heliprobe ^14^C-UBT is an easy-to-perform, rapid-response, and accurate test for *H. pylori* diagnosis, suitable for office use.

## 1. Introduction

*Helicobacter pylori* (*H. pylori*) is a spiral shaped microaerophilic gram-negative bacterium that resides in the gastric epithelial mucosa and induces an inflammatory response leading to gastritis and peptic ulcer disease [1, 2]. It has been implicated as playing a role in gastrointestinal malignancies, especially gastric adenocarcinoma and MALToma until the latter could be treated with *H. pylori *eradication [3, 4]. 

*H. pylori* has a worldwide prevalence rate of about 50%, with a higher prevalence in developing countries [5, 6]. According to population-based studies, it has been shown that the *H. pylori* infection rate is very high in the Iranian population [7].

*H. pylori* detection can be made with diverse diagnostic tests, which are technically divided into invasive and noninvasive based on whether endoscopy is required or not. Invasive tests offer the possibility of obtaining tissue samples, which can be used for a rapid urease test (RUT), culture, polymerase chain reaction (PCR), and histopathologic evaluation. Noninvasive tests include serum *H. pylori* IgG antibody titer, the urea breath test (UBT), and *H. pylori* stool antigen assay. Compared to noninvasive diagnostic modes, however, invasive techniques are inconvenient for patients and also have higher cost [8]. 

A UBT diagnostic test is based on the fact that swallowed “labeled carbon-containing urea” is broken down to ammonia and carbon dioxide (CO_2_) by the urease-producing microorganism (*H. pylori*) in the gastric mucosa, and, finally, tagged carbon within the liberated CO_2_ is detected in exhaled breath samples [9, 10].

Between two carbon isotopes (^13^C and ^14^C), which are used for the UBT, the ^13^C isotope has the difficulty of requiring more complex equipment, such as a mass spectrophotometer and administration of a pretest meal such as citric acid. However, when the ^14^C isotope is utilized, the required equipment is only a portable compact beta-scintillation counter, which offers the convenience of performing the test in the office. Although the ^14^C isotope is radioactive, microdose (1 *μ*Ci) ^14^C has the minimal radiation of one day background exposure [11].

We encountered repeatedly, dyspeptic patients, in our clinical practice, whose UBT results were not consistent with histopathology about *H*. *pylori *infection; based on Helicobacter genetic polymorphisms and differences between *H. pylori* strains in different countries, and since clinical validation of Heliprobe ^14^C-UBT has not yet been investigated in an Iranian population, we conducted a prospective study to compare Heliprobe ^14^C-UBT performance against diagnostic gold standards [12–14]. 

## 2. Materials and Methods

We studied 125 consecutive patients with dyspepsia that had been referred for upper GI endoscopy. We defined dyspepsia, based on the Rome III criteria, as having one or more of the following conditions: postprandial fullness (termed postprandial distress syndrome), early satiation (inability to finish a normal-sized meal or postprandial fullness), and epigastric pain or burning (termed epigastric pain syndrome) [15].

We considered subjects aged 15 y–75 y. We excluded patients who had been using proton pump inhibitors, H2 blockers, or any antibiotics within four previous weeks of the endoscopic evaluation. Pregnant women, patients who had a history of *H. pylori* eradication, and those with any severe cardiopulmonary disorders or debilitating or life-threatening conditions were excluded as well. Study personnel were blinded as to patient test results. The study protocol was approved by the ethics committee of the Gastrointestinal and Liver Diseases Research Center of Guilan University of Medical Sciences, and written informed consents were obtained from each participant.

After an overnight fast, patients underwent gastroscopy with a FUJINON endoscope, and four separate gastric biopsies were taken, three from the antrum and one from the corpus. One of the antral samples was used for a rapid urease test (RUT), and two antral samples plus the corpus biopsy sample were used for histopathologic examination.

To perform the RUT, we utilized a home-made liquid rapid urease kit (Gastric Urease, Bahar Afshan Co., Iran). We put tissue samples in a yellow-colored reagent liquid, and results were read after 30 min, 60 min, and finally after 24 hours. Liquid color changes into deep red, purple, or violet indicated a positive result. Negative results were indicated by no color change. Tissue samples were prepared with standard hematoxylin and eosin (H&E) and Giemsa stainings for histopathologic investigation. The histopathology exam result was considered positive when *H*. *pylori* was detected in either of the stains and negative when the organism was not detected in any.

One blood sample was obtained from each patient to be examined for anti *H. pylori *IgG titer. We utilized the ELIZA method (LDN, Nordhorn, Germany), and sera with titers that were >11 international units (IU) were considered positive (test cut-off point: 10 IU, <9 IU: negative, 9–11 IU: doubtful, >11 IU: positive); we regarded doubtful results as negative.

After gastroscopy, we performed Heliprobe ^14^C-UBT tests. In order to carry out the UBT, after an overnight fast, the patient swallowed a ^14^C-labeled urea-containing capsule (Helicap, Institute of Isotopes, Budapest, Hungary) with water. The overall activity of these capsules is as small as 1 *μ*Ci (37 KBq). After 15 minutes, the patient breathed out into a dry cartridge (Heliprobe breath card, Kibion AB, Uppsala, Sweden) through its mouthpiece until the color of the card indicator changed from orange to yellow, which took about 1 min to 2 min. Thereafter, the breath card was inserted into a small desktop Geiger Müller counter (Heliprobe Analyser, Kibion AB, Uppsala, Sweden), and the radioactivity of the breath samples was read after 250 seconds of an automated process. Finally, the test results were expressed on the LCD of the analyzer in a numeric fashion (0: patient not infected, 1: borderline result, 2: patient infected), which corresponded to radioactivity as count per minute (CPM): <25 CPM: patient not infected, 25–50 CPM: borderline result, >50 CPM: patient infected. We considered grades 0 and 1 as negative results in our study, and only samples with activities that were more than 50 CPM (expressed as no. 2 on the counter LCD) were regarded as positive.

Descriptive analysis was done for demographic features. Gold standard for *H. pylori *positivity was defined as “positive results for any two of three diagnostic methods (histopathology, RUT, serology).” Sensitivity, specificity, negative and positive predictive values (NPV and PPV), and the accuracy of the UBT were computed against our GS; for categorical variables, 95% confidence interval (95% CI) was calculated.

## 3. Results

We enrolled 125 consecutive patients in our study according to the above mentioned inclusion and exclusion criteria; 65 (52%) were females, and 60 (48%) were males. Patient ages ranged from 18 y to 66 y with a mean of 35.81 ± 12.97 y.

As a result of histopathologic evaluation of tissue specimens, 69 (55.2%) patients were found to be infected with *H. pylori, *and 56 (44.8%) were not infected. Serologic examination of patient sera samples for IgG antibody against *H. pylori *showed 87 (69.6%) positive results, while the remaining 38 (30.4%) were seronegative. The RUT results were positive in 63 (50.4%) patients and negative in 62 (49.2%) patients. *H. pylori *infection was found in 67 (53.6%) subjects by the ^14^C urea breath test (^14^C-UBT), and 58 (46.4%) subjects were negative.

All four tests were positive or negative in 57 (45.6%) and 34 (27.2%) patients, respectively. Only 2 patients had no histopathologic evidence of *H. pylori* infection, whilst their RUT and serology results were positive. (Incidentally these two patients also had positive UBTs.); We think that this might be the result of errors during sampling or histopathologic examination. 20 patients showed solitary positive serology tests which indicated recent past infection.  Table 1 shows other discordances between our test results.

Compared to our GS, the UBT could correctly detect 67 of 71 *H. pylori* infected subjects with 94% sensitivity (95% CI: 85–98%) and 100% positive predictive value (PPV; 95% CI: 93–100%). The UBT also excluded correctly all 54 uninfected patients with 100% specificity (95% CI: 92–100%) and negative predictive value (NPV) of 93% (95% CI: 82–98%). The UBT showed 97% accuracy (Table 2).

## 4. Discussion

*H. pylori* has a high prevalence rate in developing countries, such as Iran. According to data reported by Derakhshan et al., *H. pylori *must be considered a risk factor for noncardiac gastric adenocarcinoma in Iranian patient [16]. Eradication of *H. pylori *could lower the incidence rate of gastric cancer [17]. A more precise diagnosis of *H. pylori,* allowing an earlier eradicating treatment, may play a crucial role in cancer prevention strategies. Currently, there is an increasing need for an easy-to-perform, accurate, and readily available diagnostic technique in *H. pylori *prevalent populations. Considering economic concerns and the availability of tests in developing countries, as well as testing possibilities in medical clinics, we recommend use of the Heliprobe ^14^C-UBT over other noninvasive and invasive tests.

Based on the data that suggest that the combination of tests increases their overall diagnostic power, we combined *H. pylori* serologic assessment and the RUT with histopathology in order to account for the possibility of very few false negative results from microscopic examination (which were seen in two cases in our study) [18]. We discovered that the ^14^C-UBT has 94% sensitivity, 100% specificity, 100% PPV, 93% NPV, and 97% accuracy, compared with GS. These findings are compatible with Jonaitis et al.'s study results, with their defined *H. pylori* positive gold standard as “at least one positive test of RUT or histopathology”. They found that Heliprobe had 92% sensitivity, 100% specificity, 100% PPV, 84% NPV, and 94% accuracy [19]. Our results are also in accordance with Ozdemir et al.'s results for Heliprobe performance (96.6% sensitivity, 100% specificity, 93.7% PPV, 100% NPV, and 97.7% accuracy), although their *H. pylori *positive gold standard was defined as “positivity of any two of the three following tests (RUT, PCR, and histopathology)” [20].

In Rasool et al.'s study regarding histopathology alone as GS, Heliprobe sensitivity, specificity, NPV, PPV, and accuracy were 92%, 93%, 84%, 97%, and 93%, respectively, which were consistent with our results although the studies' gold standards are slightly different [21]. In another study, conducted by Öztürk et al. that is again regarding only histopathology as GS, Heliprobe had higher sensitivity (100%), but its specificity (76%) was lower than that of our study [22]. 

People who undergo medical diagnostic tests using radioisotopes are often worried about radiation exposure. The half life of the ^14^C isotope is about 5000 years, but, with regards to the short biologic half life of urea, more than two-thirds of the tagged urea will be excreted in the urine within the following three days of the test; moreover, the total dose of the ^14^C used in the test is very low, and activity of this quantity of isotope was evaluated as 1 *μ*Ci. Accordingly, based on the published data, about 800 breath testing episodes must be carried out for one person to receive an effective dose equivalent to the amount that an average person absorbs from natural sources in one year [23]. Considering the few times a person needs to be tested with the ^14^C-UBT, the lifelong cumulative radiation of the test is negligible. Even in conditions of repeated UBT testing, radiation exposure risk is very low. Previously, ^14^C-UBT was not used in children because of the concerns about the radiation hazards; however, diverse studies have established its safety in pediatric patients [11, 24]. Although no experimental study has yet been done to assess ^14^C-UBT safety in pregnancy, Bentur et al. have claimed that, in view of the insignificant ^14^C radioactivity, fetal radiation exposure is extremely lower than teratogenic thresholds [25].

One of the advantages of the Heliprobe system is that it can be used in a clinical setting, allowing the preparation of test results on-site in less than one hour. The portable beta-scintillation counter that is used in this test could simply be placed on a desktop; however, the ^13^C-UBT needs a sophisticated mass spectrophotometer to read the results. Of course, ^13^C-UBT has some advantages over ^14^C-UBT such that the former utilizes a nonradioactive isotope that makes it suitable to use in pregnant women and children. Although some studies, as mentioned earlier, have already emphasized on ^14^C-UBT safety in children, ^13^C-UBT is still the preferred method in them. Considering ^13^C safety, it is also a better isotope than ^14^C for epidemiologic studies, as some studies used it to investigate *H. pylori *routes of transmission in preschool age [26, 27].

Stool antigen is another sensitive and specific noninvasive diagnostic test for *H. pylori* [28]. Although it is competitive with the ^14^C-UBT in terms of accuracy, but it is not appropriate for office use because it is a time consuming exam regarding sampling limitations and off-site test interpretation.

Conclusively, compared to invasive gold standards, the Heliprobe ^14^C-UBT is an accurate, sensitive, and specific test for *H*. *pylori *diagnosis. The main advantages of the Heliprobe ^14^C-UBT are its rapidity and patient convenience. Furthermore, in view of the very low radioactivity of the Heliprobe ^14^C-UBT and its portability, this test seems to be a more suitable option for office use than a nonradioactive, complex and off-site ^13^C-UBT as well as other invasive diagnostic modalities.

## Figures and Tables

**Table 1:** Distribution of *H. pylori *diagnostic test discordant results.

Patients (*n*)	Histology	RUT	Serology	UBT
57	+	+	+	+
4	+	+	−	+
4	+	−	+	+
4	+	−	+	−
2	−	+	+	+
20	−	−	+	−
34	−	−	−	−

RUT: rapid urease test; UBT: urea breath test; +: positive; −: negative; *n*: number.

**Table 2:** Diagnostic performance of Heliprobe ^14^C-UBT against gold standard.

Heliprope ^14^C-UBT compared to:	Sensitivity (95% CI)	Specificity (95% CI)	PPV (95% CI)	NPV (95% CI)	Accuracy (95% CI)
Gold standard	94% (85–98%)	100% (92–100%)	100% (93–100%)	93% (82–98%)	97%

^14^C-UBT: urea breath test with labeled carbon-14; RUT: rapid urease test; PPV: positive predictive value; NPV: negative predictive value; CI: confidence interval. For gold standard definitions refer to the text.

## References

[1] Goodwin CS, Worsley BW (1993). Microbiology of Helicobacter pylori. *Gastroenterology Clinics of North America*.

[2] Papatheodoridis GV, Sougioultzis S, Archimandritis AJ (2006). Effects of Helicobacter pylori and nonsteroidal anti-inflammatory drugs on peptic ulcer disease: a systematic review. *Clinical Gastroenterology and Hepatology*.

[3] Huang JQ, Sridhar S, Chen Y, Hunt RH (1998). Meta-analysis of the relationship between Helicobacter pylori seropositivity and gastric cancer. *Gastroenterology*.

[4] Papa A, Cammarota G, Tursi A, Gasbarrini A, Gasbarrini G (2000). Helicobacter pylori eradication and remission of low-grade gastric mucosa-associated lymphoid tissue lymphoma: a long-term follow-up study. *Journal of Clinical Gastroenterology*.

[5] Pounder RE, Ng D (1995). The prevalence of Helicobacter pylori infection in different countries. *Alimentary Pharmacology and Therapeutics*.

[6] Hussein NR (2010). Helicobacter pylori and gastric cancer in the Middle East: a new enigma?. *World Journal of Gastroenterology*.

[7] Malekzadeh R, Derakhshan MH, Malekzadeh Z (2009). Gastric cancer in Iran: epidemiology and risk factors. *Archives of Iranian Medicine*.

[8] Vakil N, Rhew D, Soll A, Ofman JJ (2000). The cost-effectiveness of diagnostic testing strategies for Helicobacter pylori. *American Journal of Gastroenterology*.

[9] Chey WD (2000). Accurate diagnosis of Helicobacter pylori. 14C-urea breath test. *Gastroenterology Clinics of North America*.

[10] Öztürk E (2008). Diagnostic methods of Helicobacter pylori infection. *Gülhane Tıp Dergisi*.

[11] Leide-Svegborn S, Stenström K, Olofsson M (1999). Biokinetics and radiation doses for carbon-14 urea in adults and children undergoing the Helicobacter pylori breath test. *European Journal of Nuclear Medicine*.

[12] Chung C, Olivares A, Torres E, Yilmaz O, Cohen H, Perez-Perez G (2010). Diversity of VacA intermediate region among Helicobacter pylori strains from several regions of the world. *Journal of Clinical Microbiology*.

[13] Douraghi M, Talebkhan Y, Zeraati H (2009). Multiple gene Sstatus in helicobacter pylori strains and risk of gastric cancer development. *Digestion*.

[14] Talebkhan Y, Mohammadi M, Mohagheghi MA (2008). CagA gene and protein status among Iranian Helicobacter pylori strains. *Digestive Diseases and Sciences*.

[15] Tack J, Talley NJ, Camilleri M (2006). Functional gastroduodenal disorders. *Gastroenterology*.

[16] Derakhshan MH, Malekzadeh R, Watabe H (2008). Combination of gastric atrophy, reflux symptoms and histological subtype indicates two distinct aetiologies of gastric cardia cancer. *Gut*.

[17] Fukase K, Kato M, Kikuchi S (2008). Effect of eradication of Helicobacter pylori on incidence of metachronous gastric carcinoma after endoscopic resection of early gastric cancer: an open-label, randomised controlled trial. *The Lancet*.

[18] Thijs JC, van Zwet AA, Thijs WJ (1996). Diagnostic tests for Helicobacter pylori: a prospective evaluation of their accuracy, without selecting a single test as the gold standard. *American Journal of Gastroenterology*.

[19] Jonaitis LV, Kiudelis G, Kupcinskas L (2007). Evaluation of a novel 14C-urea breath test “Heliprobe” in diagnosis of Helicobacter pylori infection. *Medicina*.

[20] Ozdemir E, Karabacak NI, Degertekin B (2008). Could the simplified (14)C urea breath test be a new standard in noninvasive diagnosis of Helicobacter pylori infection?. *Annals of Nuclear Medicine*.

[21] Rasool S, Abid S, Jafri W (2007). Validity and cost comparison of 14carbon urea breath test for diagnosis of H Pylori in dyspeptic patients. *World Journal of Gastroenterology*.

[22] Öztürk E, Yeşilova Z, Ilgan S (2003). A new, practical, low-dose 14C-urea breath test for the diagnosis of Helicobacter pylori infection: clinical validation and comparison with the standard method. *European Journal of Nuclear Medicine and Molecular Imaging*.

[23] Stubbs JB, Marshall BJ (1993). Radiation dose estimates for the carbon-14-labeled urea breath test. *Journal of Nuclear Medicine*.

[24] Pathak CM, Kaur B, Khanduja KL (2010). 14C-urea breath test is safe for pediatric patients. *Nuclear Medicine Communications*.

[25] Bentur Y, Matsui D, Koren G (2009). Safety of 14C-UBT for diagnosis of Helicobacter pylori infection in pregnancy. *Canadian Family Physician*.

[26] Brenner H, Rothenbacher D, Bode G, Adler G (1998). Parental history of gastric or duodenal ulcer and prevalence of Helicobacter pylori infection in preschool children: population based study. *The British Medical Journal*.

[27] Vandenplas Y, Blecker U, Devreker T (1992). Contribution of the 13C-urea breath test to the detection of Helicobacter pylori gastritis in children. *Pediatrics*.

[28] Gisbert JP, Pajares JM (2004). Stool antigen test for the diagnosis of Helicobocter pylori infection: a systematic review. *Helicobacter*.

